# Characterization of mesenchymal stem cells in human fetal bone marrow by single-cell transcriptomic and functional analysis

**DOI:** 10.1038/s41392-023-01338-2

**Published:** 2023-03-31

**Authors:** Ping Zhang, Ji Dong, Xiaoying Fan, Jun Yong, Ming Yang, Yunsong Liu, Xiao Zhang, Longwei Lv, Lu Wen, Jie Qiao, Fuchou Tang, Yongsheng Zhou

**Affiliations:** 1 Department of Prosthodontics, Peking University School and Hospital of Stomatology, 100081 Beijing, China; 2 National Center for Stomatology & National Clinical Research Center for Oral Diseases & National Engineering Reasearch Center of Oral Biomaterials and Digital Medical Devices & Beijing Key Laboratory of Digital Stomatology & National Health Commission Key Laboratory of Digital Technology of Stomatology, 100081 Beijing, China; 3 Guangzhou Laboratory, 510005 Guangzhou, China; 4 Biomedical Pioneering Innovation Center and Center for Reproductive Medicine, Ministry of Education Key Laboratory of Cell Proliferation and Differentiation, 100871 Beijing, China; 5 Beijing Advanced Innovation Center for Genomics, School of Life Sciences, Department of Obstetrics and Gynecology, Third Hospital, Peking University, 100871 Beijing, China; 6 Beijing Key Laboratory of Reproductive Endocrinology and Assisted Reproductive Technology, 100191 Beijing, China; 7 Peking-Tsinghua Center for Life Sciences, Peking University, 100871 Beijing, China

**Keywords:** Cell biology, Medical research

## Abstract

Bone marrow mesenchymal stromal/stem cells (MSCs) are a heterogeneous population that can self-renew and generate stroma, cartilage, fat, and bone. Although a significant progress has been made toward recognizing about the phenotypic characteristics of MSCs, the true identity and properties of MSCs in bone marrow remain unclear. Here, we report the expression landscape of human fetal BM nucleated cells (BMNCs) based on the single-cell transcriptomic analysis. Unexpectedly, while the common cell surface markers such as CD146, CD271, and PDGFRa used for isolating MSCs were not detected, LIFR^+^PDGFRB^+^ were identified to be specific markers of MSCs as the early progenitors. In vivo transplantation demonstrated that LIFR^+^PDGFRB^+^CD45^-^CD31^-^CD235a^-^ MSCs could form bone tissues and reconstitute the hematopoietic microenvironment (HME) effectively in vivo. Interestingly, we also identified a subpopulation of bone unipotent progenitor expressing TM4SF1^+^CD44^+^CD73^+^CD45^-^CD31^-^CD235a^-^, which had osteogenic potentials, but could not reconstitute HME. MSCs expressed a set of different transcription factors at the different stages of human fetal bone marrow, indicating that the stemness properties of MSCs might change during development. Moreover, transcriptional characteristics of cultured MSCs were significantly changed compared with freshly isolated primary MSCs. Our cellular profiling provides a general landscape of heterogeneity, development, hierarchy, microenvironment of the human fetal BM-derived stem cells at single-cell resolution.

## Introduction

Bone marrow (BM)-derived mesenchymal stromal/stem cells (MSCs), which is characterized by non-hematopoietic, plastic-adherent, colony-forming cells, were first identified by Friedenstein and colleagues decades ago. Despite their complexity and heterogeneity, evidences increasingly support the notion that MSCs exhibit trilineage differentiation potentials and represent an important component of the hematopoietic stem cell (HSC) niche in bone marrow (BM).^1–4^ Many studies have expanded the knowledge of MSCs through the use of genetically modified mice. However, due to the technical and material limitations, little is known about the cellular biology of human bone marrow mesenchymal cells. Furthermore, the development of MSCs during embryonic stages is also an unresolved issue. It is particularly important to study the development of MSCs, which, in turn, could help us better understand the physiological functions of MSCs.

The identification of MSC marker genes is crucial for isolating and characterizing MSCs in vivo. Matsuzaki group first identified MSCs based on the expression of the PDGFRa and Sca-1 in the non-hematopoietic and endothelial compartment in the bone marrow of adult mice.^2^ By using transgenic mouse model, Nestin, Leptin-Receptor (LepR) and Grem1 expressing cells were identified with MSC/skeletal stem cell (SSC) activity and represent the major source for HSC niche factors such as *CXCL12* and *SCF* in the bone marrow.^5–7^ In contrast, human MSCs have long been identified through the flow cytometry analysis by using monoclonal antibodies. CD146^+^ MSCs from human bone marrow were observed to enrich for CFU-Fs and be able to generate the hematopoietic bone marrow.^8^ CD271 was another broadly accepted surface marker of MSCs from human bone marrow, CFU-Fs were highly and exclusively enriched in both CD271^+^CD146^-/low^ and CD271^+^CD146^+^ cells.^9–11^ Other surface markers including CD105, CD73, CD90, CD49a, CD140b, MSCA-1, SSEA-4, and STRO-1 were also used to sort human MSCs alone or in combination.^12^ Even though large amount of information about human MSCs has been obtained based on experiments performed in culture, the complexity and physiological characteristics of these cells in vivo remains poorly investigated.

Most recently, Huelsken’s group showed that when primary BM-derived stem cells (BMSCs) were expanded in vitro, their capacity to support hematopoiesis was significantly lost.^13^ Another report demonstrated that primary murine MSCs exhibited high homing efficiency to the BM, but lose homing ability after in vitro culture.^14^ Mx-1-Cre mice-derived cells possess CFU-Fs activity and trilineage differentiation potential in vitro, but only show a limited differentiation ability in vivo.^15^ CD146 has been reported to label human BMSCs in vivo, however, its expression level is significantly changed when the cells are cultured.^9^ Moreover, significant differences were also identified in global DNA methylation profiles of BMSCs following cell expansion.^16^ All these studies clearly demonstrated that the in vitro features of MSCs/SSCs could not faithfully reflect their in vivo function. Unfortunately, our present knowledge of human BMSCs is mainly advanced by in vitro culture systems. Thus, strategies to identify the real identity of human BMSCs/SSCs in vivo would be of great help for clearing up the “stem-cell mess of MSCs”.^17^

**Fig. 1:**
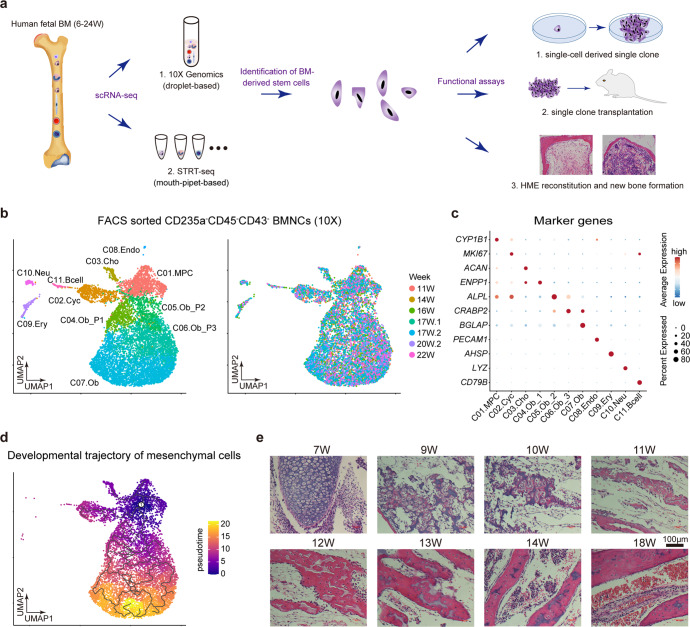
Expression landscape of human fetal BM stromal cells. **a** Diagrammatic sketch of the current study. **b** UMAP showing the clustering (left) and week information (right) of all FACS-sorted CD235a^-^CD45^-^CD43^-^ nucleated cells sampled from the human fetal bone marrow, which were sequenced by 10x Genomics scRNA-seq technique. **c** Dotplot showing the expression patterns of representative marker genes in each major cluster identified in Fig. 1b. The color key from blue to red indicates low to high expression levels, respectively. Dot size indicates the percent of cells expressing a certain gene. **d** Developmental trajectory of mesenchymal cells inferred by monocle 3 algorithm. **e** H&E staining of femur sections from different embryonic stages

The recent breakthrough of single-cell RNA sequencing (scRNA-seq) technique allows us to study the freshly isolated primary cells at single-cell resolution, and investigate the bona fide identity of human MSCs/SSCs in vivo.^18^ On the other hand, based on the transcriptome of each cell, we could better understand the complexity and heterogeneity of MSC/SSC populations.^19^ In the present study, we performed a comprehensive screening of human fetal BM nucleated cells (BMNCs) at single-cell resolution (Fig. 1a). We isolated primary BMNCs from 46 human embryos ranging from 6 to 24 developmental weeks and performed scRNA-seq analysis using two complementary strategies (STRT and 10x Genomics) to balance between accuracy and throughput. Based on systematic bioinformatics analyses and experimental validations, we identified two types of stem cells in the human BM. This current work would enhance our understanding about human fetal BM-derived MSCs and their heterogeneity and microenvironment in vivo.

## Results

### Expression landscape of human fetal BM stromal cells

In order to explore the cellular diversity of the human fetal BM stroma, we performed single-cell transcriptomic profiling of BMNCs using 10x Genomics scRNA-seq technique (Supplementary Table 1). Since the BM was fulfilled with red blood cells, we lysed these red cells by ice-cold sterile H_2_O.^20^ To test the feasibility of our experimental protocol, 2634 single cells passing the quality control were obtained from two embryos (i.e., 20- and 21-week-old). In total, we identified 12 clusters with batch effect correction in Harmony and unsupervised clustering in Seurat (Supplementary Fig. 1a).^21,22^ According to the classic marker genes, these clusters were annotated as: two clusters of erythrocytes, specifically expressing *GYPA* and *HBG1*, respectively; basophils (*CSF2RB*); myeloid cells (*PLEK*); neutrophils (*AZU1*); monocytes (*CSTA*); natural killer cells (*SPINK2*); three clusters of B cells, highly expressing *CD79A*, *LTB* and *JCHAIN*, respectively; macrophages (*CSF1R*); and mesenchymal cells, expressing collagen triple helix repeat containing 1 (*CTHRC1*) (Supplementary Fig. 1b). As indicated above, by progressive depletion of the red cells with instantaneous H_2_O treatment, the majority of BMNCs at late developmental stages (20–21 weeks) are hematopoietic cells.

To capture relatively rare BM stromal cells, we next performed scRNA-seq on the sorted non-hematopoietic CD235a^-^CD45^-^CD43^-^ cells (Supplementary Fig. 1c), and obtained 8,725 sorted cells from 9 embryos (11–22 weeks). As shown in Fig. 1b, the human fetal BM CD235a^-^CD45^-^CD43^-^ cells were divided into 11 clusters. Based on the differentially expressed genes (DEGs) and enriched gene ontology (GO) terms (Fig. 1c, Supplementary Fig. 1d, e, and Supplementary Table 2), we annotated them as seven mesenchymal clusters, three hematopoietic clusters and one endothelial cluster. Most of the sorted cells were non-hematopoietic cells, indicating the accuracy of FACS sorting. Among the seven mesenchymal clusters, we identified four clusters exhibiting a developmental trajectory of osteoblast (OB) lineage, from OB progenitors to mature OBs (OB-P1, OB-P2, OB-P3, and OB). We also detected a cluster with chondrocyte lineage (specifically expressing *ACAN*) and a cluster of cycling mesenchymal cells. Importantly, we found a cluster of mesenchymal progenitor cells with no distinct differentiation characteristics, highly expressing *CYP1B1*, which we designated as mesenchymal progenitor cells (MPC) (Fig. 1c). This result was also supported by the developmental trajectory inferred by Monocle (Fig. 1d).^23^ In addition, we did not detect any adipocytes in either the single-cell data or H&E and Oil-red O staining of femur sections (Fig. 1e and Supplementary Fig. 1f), which demonstrated the exiguity of adipocytes in human fetal BMs.

### Heterogeneity of MSCs in human fetal BM

**Fig. 2:**
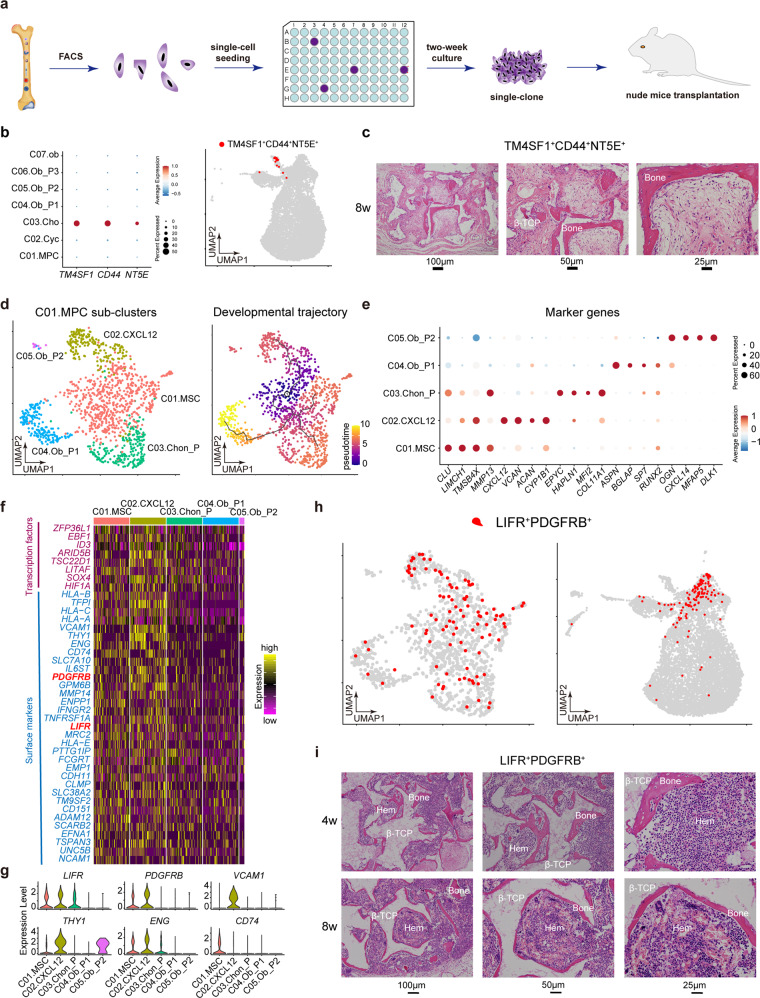
Heterogeneity of MSCs in human fetal BM. **a** Study overview for nude mice transplantation. **b** Dotplot (left) and UMAP (right) showing the specific expression of gene combination of *TM4SF1*, *CD44* and *NT5E* in chondrocytes. The color key from blue to red indicates low to high expression levels, respectively. Dot size indicates the percent of cells expressing a certain gene. **c** Abundant new bones were formed after 8 weeks transplantation of single clone derived from single primary TM4SF1^+^CD44^+^CD73^+^/CD45^-^CD31^-^CD235a^-^ cell. **d** UMAP showing the clustering result (left) and developmental trajectory (right) of MPCs identified in Fig. 1b. **e** Dotplot showing expression levels of representative marker genes in each cluster identified in Fig. 2d. **f** Heatmap showing the differentially expressed transcription factors and surface markers of C01.MSC and C02.CXCL12. The color key from purple to yellow indicates low to high expression levels, respectively. **g** Violin plots showing the expression levels of potential surface markers for C01.MSC and C02.CXCL12. **h** UMAP showing the specific expression of gene combination of *LIFR* and *PDGFRB* in C01.MSC and C02.CXCL12. **i** Hematopoietic cell clusters (hem) appeared at 4 weeks and matured at 8 weeks after transplantation of single clone derived from single primary LIFR^+^PDGFRB^+^/CD45^-^CD31^-^CD235a^-^ cell. Abundant new bones were formed after 4 weeks transplantation. β-TCP: hydroxyapatite carrier

A recent study clarified that perinatal chondrocytes form most of the new osteoblasts and decreased progressively with age.^24^ Thus, we experimentally tested the chondrocyte cluster by individually seeding the sorted single cells into 96-well plates separately, and then subcutaneously implanting expanded colonies into the dorsal side of the nude mice with β-TCP carrier (Fig. 2a). As shown in Fig. 2b, the chondrocyte cluster exclusively expressed surface markers *CD44*, *NT5E*, *TM4SF1*, we subsequently harvested CD44^+^CD73^+^TM4SF1^+^/CD45^-^CD31^-^CD235a^-^ cells through FACS sorting (Supplementary Fig. 2a). STRT analysis further confirmed the accuracy of FACS (Supplementary Fig. 2b). We found that the colonies expanded from single CD44^+^CD73^+^TM4SF1^+^ cells were able to form bony tissues effectively (Fig. 2c). Therefore, our study was consistent with the recent finding that perinatal chondrocytes can give rise to new osteoblasts.^24^ We further sorted the osteo-progenitor cells (OB_P1, P2, P3) using FACS antibody ENPP1 and ANKH. However, single clones derived from single ENPP1^+^/ANKH^+^ cells failed to form new bone (data not shown).

MSCs in the bone marrow have been defined as multipotent stem cells that can differentiate into osteocytes, adipocytes, and chondrocytes,^25,26^ however, their exact identity in vivo remains unclear. We subsequently distinguished MPCs into five major subsets by sub-clustering and developmental trajectory analysis, namely MSCs (C01.MSC), CXCL12^+^ MSCs (C02.CXCL12), chondrocyte progenitors (C03.Chon-P) and two OB progenitor sub-clusters (C04.Ob-P1 and C05.Ob-P2) (Fig. 2d, e). In particular, one subset significantly expressed the key functional osteoblast specific gene *ASPN* and *BGLAP* (C04.Ob-P1); the other subset highly expressed *EPYC* and *HAPLN1*, which we annotated as chondro-progenitors (C03.Chon-P). In fact, the osteo-progenitors and chondro-progenitors both can be derived from MSCs. To experimentally validate the MSCs, we first choose effective surface marker. As shown in Fig. 2f–h, *LIFR* combined with *PDGFRB* exhibited good pattern for C01.MSC and C02.CXCL12. Compared with *LIFR*, the expression level of *ENG* in C01.MSC was lower. We next validated these two MSC clusters by sorting LIFR^+^PDGFRB^+^/CD45^-^CD31^-^CD235a^-^ cells for nude mice transplantation (Fig. 2i and Supplementary Fig. 2c). STRT scRNA-seq analysis first verified the accuracy of FACS (Supplementary Fig. 2d). As described above, single LIFR^+^PDGFRB^+^/CD45^-^CD31^-^CD235a^-^ cell was seeded into the 96-well plate, and after 2 weeks, single clones were harvested and transplanted into the dorsal side of nude mice. As shown in Fig. 2i, we observed abundant new bony ossicles 4 weeks after transplantation. And 8 weeks after transplantation, hematopoiesis which is reminiscent of those found in the bone marrow apparently appeared.

### Discrepancy between MSCs at early and late developmental stages

**Fig. 3:**
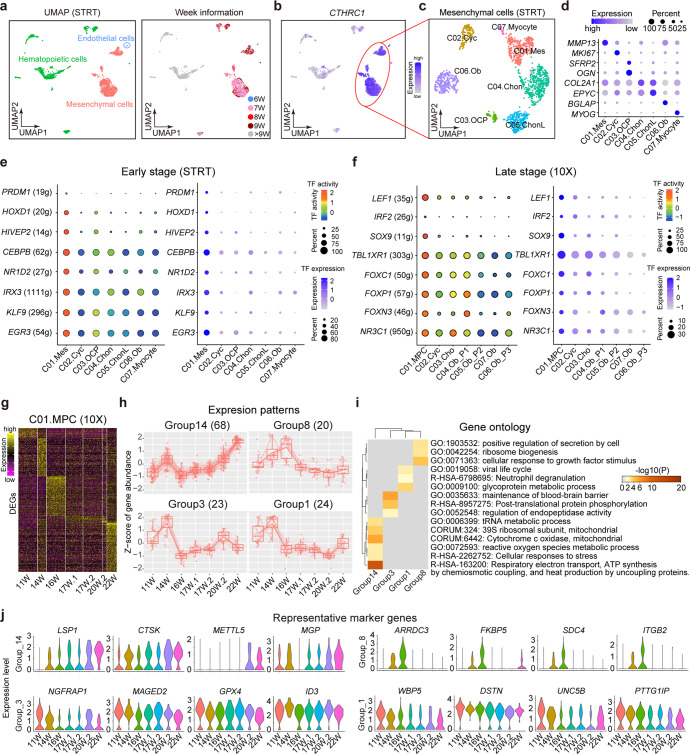
Discrepancy between MSCs at early and late developmnental stages. **a** UMAP showing the three major groups (left) and their developmental stages (right) of all randomly picked fresh cells from human fetal BM at early stages. **b** UMAP showing the specific expression of *CTHRC1* in mesenchymal cells. **c** UMAP showing the 7 clusters of mesenchymal cells at early stages. **d** Dotplot showing the expression levels of representative marker genes in each cluster identified in Fig. 3c. The color key from light blue to dark blue indicates low to high expression levels, respectively. Dot size indicates the percent of cells expressing a certain gene. **e** TFs that were specifically activated and expressed during the early developmental stages. **f** TFs that were specifically activated and expressed during the late developmental stages. **g** The development of MSCs at late stages identified in Fig. 2d. Heatmap showing the DEGs of MSCs at each stage. The color key from purple to yellow indicates low to high expression levels, respectively. **h** Selected expression patterns along the development of MSCs. The gene number is in the bracket. **i** Enriched terms using the genes of each expression pattern along the development of MSCs. **j** Violin plots showing the expression levels of marker transcription factors along the development of MSCs

In order to trace the origin of human fetal MSCs, we tried to capture mesenchymal cells at earlier developmental stages (6–9w). However, due to the limited numbers of cells acquired at early developmental stages, instead of using 10x Genomics scRNA-seq technique that is suitable for capturing 1000–10,000 single cells per sample, we harvested cells via random picking and sequenced them using STRT scRNA-seq technique. In total, 2,989 high-quality single-cell transcriptomes of 17 embryos ranged from 6 to 24 weeks were captured (Fig. 3a).

We identified three major groups in the STRT dataset: namely, endothelial cells (with *CDH5* specifically expressed), mesenchymal cells (highly expressing *CTHRC1*, collagen triple helix repeat containing 1), and hematopoietic cells (highly expressing *PTPRC* and *GYPA*) (Fig. 3b and Supplementary Fig. 3a, b). Notably, most of the sequenced cells at later developmental stages (after 9 weeks) were hematopoietic cells, which was consistent with 10x dataset (Supplementary Fig. 1a). The mesenchymal cells were further divided into 7 clusters (Fig. 3c). Based on the DEGs, we annotated them as mesenchymal progenitor cells (C01.Mes, which highly expressed *MMP13*); cycling progenitor cells (C02.Cyc, which were characterized by the expression of *MKI67*); osteo-chondrogenic progenitors (C03.OCP, with *OGN* and *SFRP2* upregulated); chondrocyte progenitor cells (C04.Chon, where *COL2A1* was highly expressed); late mature chondrocytes (C05, ChonL, highly expressed *EPYC*); osteoblasts (C06.Ob; with bone related gene *BGLAP* specifically upregulated); and myocytes (C07.Myocyte, specifically expressed *MYOG*) (Fig. 3d).

Transcription factors (TFs) play critical roles during a variety of biological processes. We used SCENIC to infer the gene regulatory networks from our scRNA-seq data and identify key TFs during the development of MSCs.^27^ In human fetal BM mesenchymal cells of early developmental stages, we found that *PRDM1*, *HOXD1*, *HIVEP2*, *CEBPB*, *NR1D2*, *IRX3*, *KLF9*, *EGR3 etc*. may play important roles in C01.Mes (Fig. 3e). Both the gene-expression levels and regulatory activity were specifically high in C01.Mes. However, the situation is quite different from the MSCs at later stages (11w-22w), where TFs such as *LEF1*, *IRF2*, *SOX9*, *TBL1XR1*, *FOXC1*, *FOXP1*, *FOXN3*, and *NR3C1* etc., were highly expressed and activated (Fig. 3f).

In 10x dataset, we obtained BMSCs from 7 embryos ranging from 11 to 22 weeks, which provided a chance to study their development. No significant change was detected in the cell type ratio from 11 to 22 weeks (Supplementary Fig. 3c). Next, we performed differential expression analysis for the BMSCs based on their developmental weeks. As shown in Fig. 3g, BMSCs of different development weeks exhibited their unique expression patterns, indicating a continuous development of BMSCs from 11 to 22 weeks. We next clustered the expressed genes of BMSCs into 11 groups based on their gene-expression patterns (Supplementary Fig. 3d). Among these groups, expression levels of group14 genes showed an increasing trend, expression levels of group1 and group3 genes exhibited a decreasing trend, and expression levels of group8 genes were upregulated and reached a peak at 16 weeks and then were down-regulated until 22 weeks (Fig. 3h). These four groups of genes took part in different biological processes, which might play important roles in the development of BMSCs (Fig. 3i, j).

### Cell–cell communications in the human fetal BM

**Fig. 4:**
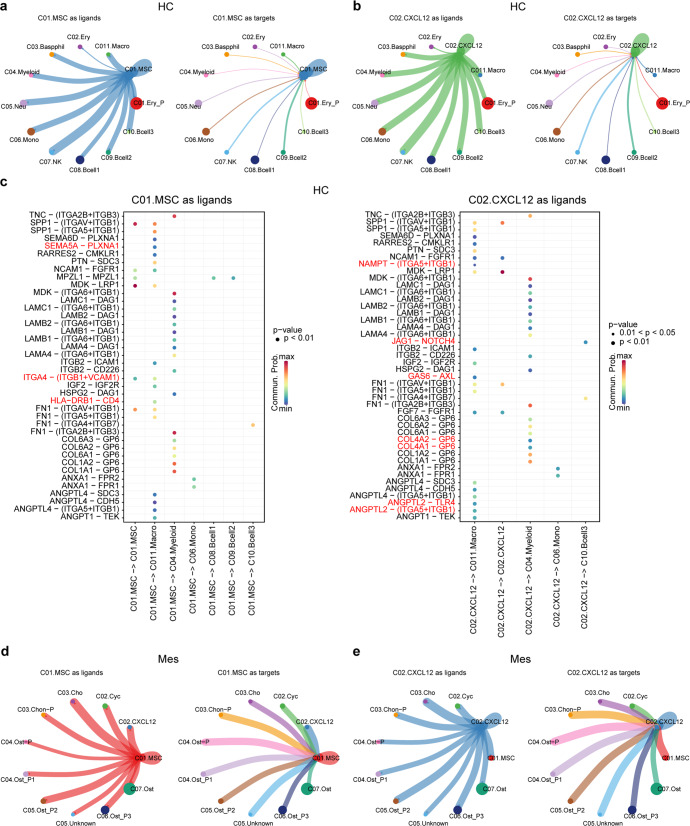
Cell–cell communications between MSCs and niche cells. **a** Cellular interaction between C01.MSC and hematopoietic cells (HC). **b** Cellular interaction between C02.CXCL12 and hematopoietic cells (HC). **c** Unique ligand-receptor pairs between MSCs and hematopoietic cells. The red ligand-receptor pairs indicate the differences between C01.MSC and C02.CXCL12. **d** Cellular interaction between C01.MSC and mesenchymal cells (Mes). **e** Cellular interaction between C02.CXCL12 and mesenchymal cells (Mes)

Investigating intercellular communications has greatly increased our understanding of stem cell-niche interactions. To fully understand the microenvironment of MSCs in the human fetal BM, we first elucidated the cellular interactions between MSCs and hematopoietic cells (HC). When MSCs were regarded as ligand, we found that most of the HCs, including basophils, myeloid cells, neutrophils, monocytes, natural killer cells, B cells, and macrophages have close ties with MSCs (Fig. 4a and Supplementary Table 3). Indeed, MSCs were highly enriched in the critical factors of HSC maintenance, such as cytokines chemokine ligand 12 (CXCL12) and stem cell factor (SCF), and CXCR4 was used as the receptor by all the HC cell types (Supplementary Fig. 4).^28–31^ Conversely, when MSCs were regarded as targets, we observed that all hematopoietic cells do not interact closely with MSCs (Fig. 4a). When MSCs were regarded as ligands, active ligand-receptor pairs were overtly detected in cell–cell communications. Like MSCs, CAR cells interact with hematopoietic cells in the same way (Fig. 4b).

We also identified unique interactions for each HC cell type with MSCs (Fig. 4c). For example, only myeloid cells expressed ITGA2B and ITGB3 as the receptors for the ligand TNC sent by MSCs; while ANGPTL1 and ANGPTL4 sent by MSC could only be received by macrophages by expressing SDC3, CDH5, (ITGA5 and ITGB1) and TEK as the receptors. Besides, MSCs and CAR cells also had unique interactions with HCs. For example, we found that MSCs could interact with macrophages and themselves through ligand-receptor pair, ITGA4–(ITGB1 + VCAM1). Previous studies indicated that VCAM1 could interact with integrin alpha-4/beta-1 (ITGA4/ITGB1) to mediate both adhesion and signal transduction, and VCAM1^+^ macrophages regulate hematopoietic stem and progenitor cells (HSPCs) homing to the vascular niche in an ITGA4 dependent manner.^32^ Thus, the VCAM1^+^ macrophages might also regulate fetal MSCs homing to the vascular niche, and both VCAM1^+^ MSCs and macrophages might play important roles in HSPCs homing in the late developmental stages. On the other hand, CAR cells secreted ANGPTL2 to attract and activate macrophages by the receptor TLR4 to produce inflammatory cytokines.^33^ We next explored the cellular interactions between MSCs and mesenchymal cells. As shown in Fig. 4d, e, whether MSCs were used as ligands or targets, their interactions with other stromal cells were quite strong. The same situation existed between CAR cells and mesenchymal cells.

### CFU-Fs in human fetal BM

**Fig. 5:**
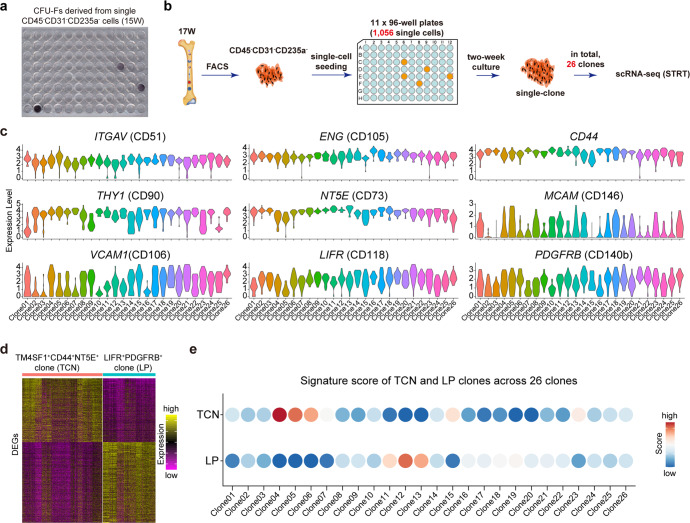
CFU-F activity of human fetal BM mesenchymal cells. **a** CFU-Fs from human fetal BM CD45^-^CD31^-^CD235a^-^ cells. **b** Study overview of the CFU-F assay of human fetal BM CD45^-^CD31^-^CD235a^-^ cells. **c** Violin plots showing the expression levels of representative surface marker genes in all single clones derived from single CD45^-^CD31^-^CD235a^-^ cells. **d** Heatmap showing the DEGs between TCN clones and LP clones. The color key from purple to yellow indicates low to high expression levels, respectively. **e** Signature score of TCN and LP clones across all 26 clones derived from single CD45^-^CD31^-^CD235a^-^ cells

To assess the CFU-F activity of mesenchymal cells, we seeded single freshly sorted CD31^-^CD45^-^CD235a^-^ BMNCs from a 15-week embryo into a 96-well plate, and detected 3 expanded clones after culture for 2 weeks (Fig. 5a). To further evaluate the CFU-F ratio, 1056 freshly sorted cells from embryos of a 17-week twins were cultured after single-cell seeding in eleven 96-well plates, and 26 colonies were observed after 2-week culture (Fig. 5b). These colonies were then digested and subjected to scRNA-seq analysis. Strikingly, a number of markers that have previously been shown to mark MSCs, such as *THY1*, *ENG*, *NT5E*, *CD44*, *ITGAV*, were found to be highly expressed in all clones. The expression level of melanoma cell adhesion molecule (*MCAM*), also known as CD146, was very different in these clones, it is highly expressed in some clones and almost not expressed in others. Notably, low-affinity nerve growth factor receptor (LNGFR/CD271), which has been described to label uncultured multipotent MSCs,^34^ was not expressed in all 26 clones (data not shown).

We wondered among these 26 clones how many clones were derived from the BM-derived stem cells. Thus, we examined single clones derived from sorted single TM4SF1^+^CD44^+^NT5E^+^ and LIFR^+^PDGFRB^+^ cells using STRT-seq analysis, which exhibited different expression patterns (Supplementary Fig. 5a, b). We then used the DEGs between LIFR^+^PDGFRB^+^ and TM4SF1^+^CD44^+^NT5E^+^ clones as their own signature genes to calculate the signature score across these 26 clones derived from the whole BMNCs, and found only a small fraction of CFUs were inferred to come from the above two stem cell populations (Fig. 5d, e). Indeed, genuine single stem cell within the BM could form clone, but the reverse statement is not valid, since only a partial of CFU-Fs are multipotent when transplanted in vivo.^26^

### Comparison between primary MSCs and cultured MSCs

**Fig. 6:**
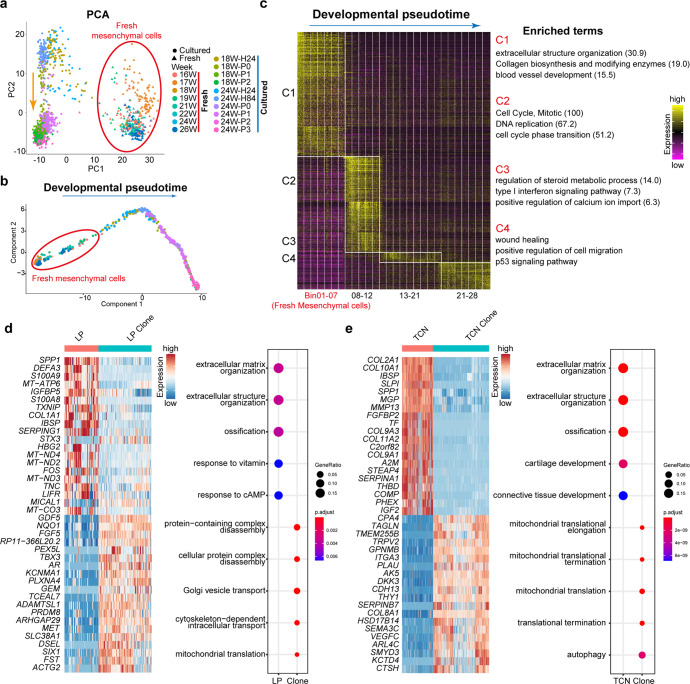
Comparison between primary and cultured BM mesenchymal cells. **a** PCA plot showing relationship between primary and cultured BM mesenchymal cells. **b** Developmental pseudotime of primary and cultured BM mesenchymal cells. **c** Cells were split into 28 bins along the developmental pseudotime. Each bin contained 50 cells and the DEGs of each bin were shown by the heatmap. All bins were classified into four major clusters according to their expression patterns. And the enriched terms using all the DEGs in each cluster were listed on the right. The number in the bracket indicates –log10 (*P*-value). **d** DEGs (left) and GO terms (right) showed that the fresh LP cells and LP clones exhibited high heterogeneity. **e** DEGs (left) and GO terms (right) showed that the fresh TCN cells and TCN clones exhibited great high heterogeneity

Previous studies have suggested that MSCs gradually lose proliferative capacity and secretive properties during expansion.^35,36^ We next made comparison between fresh and cultured BMNCs to score possible changes. Since the cultured cells were sampled from the embryos of 18 and 24 weeks, we combined fresh BMNCs sampled from the embryos of similar developmental stages (16–26 weeks) with cultured cells to perform subsequent analyses. The first axis of PCA separated fresh and cultured cells, while the second axis ordered cultured cells along the culture stages, which indicated drastic changes of the gene-expression patterns during in vitro culture (Fig. 6a). The developmental pseudotime analysis through Monocle also sorted fresh and cultured cells along the culture stages (Fig. 6b). We split all these cells into 28 bins along the inferred developmental pseudotime, each bin contained 50 cells (Fig. 6c and Supplementary Fig. 6a). We next performed DEG analysis for each bin and found that these cells could be divided into 4 major clusters (C1–C4) based on their gene-expression patterns (Fig. 6c). DEGs in each cluster demonstrated a general proliferation and differentiation process. C1 was composed of fresh mesenchymal cells, and the DEGs of C1 were predominantly associated with extracellular structure organization, collagen biosynthesis and blood vessel development, which suggested the features of fresh mesenchymal cells. Cells in C2 underwent an active proliferation period and then were followed by differentiation in C3. Unexpectedly, C4 are characterized by the activation of p53 signaling pathway, which might reflect a senescent state of MSCs with higher passages.

We further compared the freshly isolated LIFR^+^PDGFRB^+^ cells with single LIFR^+^PDGFRB^+^-derived single clones, TM4SF1^+^CD44^+^NT5E^+^ cells with their clones, respectively. During the culture of both these two cell types, genes related to extracellular matrix organization, extracellular structure organization and ossification were down-regulated, while mitochondrial translation related genes were upregulated. Besides, fresh TM4SF1^+^CD44^+^NT5E^+^ cells and cultured clones displayed much stronger discrepancy compared with the culture of primary LIFR^+^PDGFRB^+^ cells (Fig. 6d, e). Furthermore, it is important to mention that the expression of *LIFR* was lost when cultured in media, which was quite different from the significant increase of *NT5E*, *THY1* and *ENG* after long time culture (Supplementary Fig. 6b).

## Discussion

**Fig. 7:**
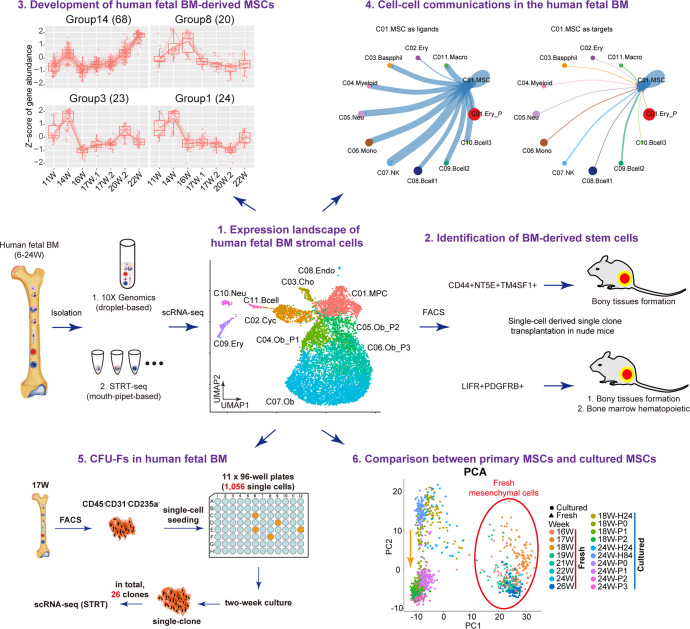
A mechanism and illustration picture of this study

A fundamental question in the study of MSCs is that the bona fide identity of human MSCs in vivo is not well defined.^37^ In the past few decades, considerable progress has been made in defining and characterizing murine bone marrow MSCs using genetically engineered mice.^6^ In addition, scRNA-seq technology helped us to gain a global recognition of the heterogeneous populations in the murine BM,^18,19^ however, the cell census of human fetal BM remains underestimated owing to material limitations. In this study, more than 10,000 human embryonic single BMNCs were subjected for scRNA-seq analysis, our cellular profiling and functional analysis not only provide a definitive answer for this question, but also broaden our understanding of the landscape of heterogeneity, development, the microenvironment of the human fetal BMSCs at single-cell resolution (Fig. 7).

Another critical question about the MSC-based bone tissue engineering is the necessity to expand the cells prior to use. In the present study, gene-expression profiles indicated an apparent gap between fresh MSCs and cultured MSCs (Fig. 6). The explicit alteration of fresh cells following ex vivo expansion, as well as the demonstration of in vivo niche for the MSCs, would be important hints for guiding us to identify optimal culture conditions for maintaining the original characteristics of in vivo MSCs. In vitro expansion that could preserve the original disposition and progenitor function of MSCs would be a great help for MSC-based bone regeneration. We also examined the CFU-F activity of human fetal BMNCs (Fig. 5). Stromal cells with CFU-F activity and multi-lineage differentiation ability in vitro were generally identified as MSCs. However, many bone marrow stromal cells have the ability of multi-lineage differentiation potential and CFU-F activity in vitro, but failed to form new bone when transplanted in vivo. On the other hand, the expression pattern of many surface markers, which are generally used for sorting MSCs displayed a great difference before and after culture. Both of which further suggested that the characteristics of MSC in vitro are not fully indicative of in vivo function.

Despite the beneficial effects of MSC therapy both in pre-clinical and clinical trials, more and more studies have produced mixed results regarding their therapeutic efficiency. The great changes between freshly isolated MSCs and cultured cells may suggest a possible explanation for the discrepancy between expected and actual results of MSC therapy. In the present study, gene-expression profiles indicated an apparent gap between primary MSCs and cultured MSCs. The fresh MSCs were endowed with multiple functions including extracellular structure organization, collagen biosynthesis and blood vessel development, which were compatible with the characteristics of MSCs. In contrast, the cultured MSCs underwent the ‘active proliferation–differentiation–senescence’ processes. Of note, the signature of fresh MSCs is quite different from that of culture-expanded MSCs (Fig. 6). In vitro expansion that could preserve the original disposition and progenitor function of MSCs would be a great help for MSC therapy.

In a recent study,^38^ Chan et al. defined a cluster of skeletal stem cells (SSCs) in human embryos, which could differentiate into cartilage and bone/stroma. We downloaded their scRNA-seq dataset and integrated it with our dataset. However, compared with our current observation of MSCs in human fetal BM, there was no specific cell population that specifically expressed the identified MSC markers in their dataset (Supplementary Fig. 7a, b), indicating the different lineages between the MSCs identified in this study and the SSCs they found.

Chiara et al. investigated the effect of isolation methods on cell populations in the scRNA-seq experiments.^18^ They analyzed the comparison between flushing of undigested BM and crushing of whole bones with enzymatically digestion, and demonstrated that only after strong physical treatment or enzymatic digestion can several populations be detected. In addition, Ninib et al. also demonstrated different murine cellular composition between bone and bone marrow.^19^ These observations in the murine BM might explain the discrepancy of human fetal SSCs releasing from enzymatically digested bones and our human embryonic MSCs from undigested flushing BM. Besides, we also compared our fetal MSCs with BM-derived MSCs from healthy adult donors cultured in the presence/absence of IFN-γ and TNF-α, which resulted in MSC-“licensing”.^39^ Although there were significant differences in distinct expression patterns among fetal, licensing^+^ and licensing^-^ MSCs, fetal MSCs did shared several immune features with licensing^+^ MSCs, such as cytokines signaling in immune system, regulation of immune response, and cellular response to cytokine stimulation (Supplementary Fig. 7c–e).

## Materials and methods

### Ethics statement

Our study was approved by the Reproductive Study Ethics Committee of Peking University Third Hospital (2012SZ-013 and 2017SZ-043). Each donor signed an informed consent form, and the study was carried out according to the ISSCR guidelines.

### Isolation of bone marrow cells

Human fetal long bones (femur and tibia) were separated, gently removed soft tissue with paper towels. Then the bone marrow was flushed with syringes, cells from the bone marrow were harvested without enzymatic digestion. Since the bone marrow was full of red blood cells, we lysed the red blood cells in the suspension by adding cold sterile H_2_O for 6 s. After 6 s lysis, immediately add 1× phosphate-buffered saline (PBS) with 4% (vol/vol) fetal bovine serum (FBS) to stop the reaction. As there are too many red blood cells in the bone marrow, transient H_2_O treatment needs to be repeated several times. After the red cells were lysed, the remaining cells were filtered into a collection tube through a 40 mm filter, and then combined into a sample for next experiment.

### Mouth-pipet-based scRNA-seq library construction

We prepared 2.5 µl of single-cell lysis buffer containing 0.8 U/µl Recombinant RNase Inhibitor (Takara, Cat.2313B), 0.38% Triton-X 100 (Sigma, Cat. T8787), 2 mM dNTP mixture (Takara, Cat.4019) and 300 nM RT primer. Ninety-six types of barcoded sequences (6-bp barcode) served as RT primers for each cell and corresponded to one barcode (Supplementary Table 4). Single cells were transferred to lysis buffer in 0.2 ml PCR tubes via mouth pipetting. The selected cells were either stored at –80 °C or directly reverse-transcribed and amplified. The single-cell transcriptome amplification steps were conducted according to STRT-seq,^40,41^ with a few modifications of the RT primers. After amplification, the cDNAs of different barcodes were combined together and purified using the DNA Clean and Concentration Kit (ZYMO, Cat. D5044) to remove free primers and primer dimers. A second round of amplification was then conducted with biotin primers containing the Illumina read2 primer sequence and indexes. Thus, after 4 cycles of PCR, the cDNAs were further fragmented using Covaris S220, and the 5’ portion of the first-strand cDNA was enriched using C1 streptavidin beads (Invitrogen, Cat. 65002). Further library construction was conducted using KAPA Hyper Prep Kits for Illumina (Cat.KK8505) following the manual. Each single-cell was designed for 0.5 G data on the Illumina HiSeq 4000 platform using 150-bp paired-end reads.

### Droplet-based scRNA-seq library construction

Cells were centrifuged at 500 × *g* for 5 min at 4 °C. Then we removed the supernatant and washed the cell pellet once with 0.04% BSA/PBS. Before loading onto the 10x Genomics Chromium chip, we calculated the concentration under the microscope. Reverse transcription, cDNA amplification and library construction were performed using the 10x Genomics Single-Cell v2 kit according to the manuals. Each library was sequenced on Illumina hiseq4000 to acquire a sequencing saturation over 90%.Cells were centrifuged at 500 × *g* for 5 min at 4 °C. Then we removed the supernatant and washed the cell pellet once with 0.04% BSA/PBS. Before loading onto the 10x Genomics Chromium chip, we calculated the concentration under the microscope. Reverse transcription, cDNA amplification and library construction were performed using the 10x Genomics Single-Cell v2 kit following the manuals. Each library was sequenced on Illumina hiseq4000 to acquire a sequencing saturation over 90%.

### Processing of scRNA-seq data

For 10x dataset, we used Cell Ranger 2.2.0 with default mapping parameters to process the raw data. Reads were consistent with the human GRCh38 genome.

For STRT dataset, we used UMI-tools^42^ to extract the barcode and unique molecular identifiers (UMIs) from R2 reads. We removed template switch oligo and polyA tail sequences from the obtained readings. The clean reads were consistent with the human GRCh38 genome using STAR.^43^ We used featureCounts^44^ to count unique mapped reads and quantified the UMIs with UMI-tools.

After obtaining the UMI expression table, we removed cells with less than 1000 detected genes and 10,000 detected transcripts from the STRT dataset. For the 10x dataset, we removed cells with fewer than 200 detected genes. Cells with high mitochondrial gene-expression fractions were also removed. We performed clustering using the Seurat package (version 2.2)^45^ (for more details, please see http://satijalab.org/seurat/). Briefly, highly variable genes were selected to perform dimensionality reduction. We used Harmony to reduce the batch effect arising from embryos’ differences, (https://github.com/immunogenomics/harmony).^21^ A graph-based clustering method in Seurat was used to determine the final clustering.

### Differentially expressed gene (DEG) analysis and gene ontology enrichment analysis

We employed Seurat to perform the DEG analysis. We used FindAllMarkers to identify DEGs for each cluster and the FindMarkers function in Seurat to identify DEGs for two given clusters. For STRT data, genes with fold-change >2 or <0.5 and adjusted *P*-value < 0.01 were regarded as DEGs; while for 10x Genomics data, default parameters (logfc.threshold = 0.25; return.thresh = 0.01) were used. Heatmaps were plotted in Seurat or the pheatmap package; violin plots were established using the Seurat package; and bar plots were established in R. Gene ontology enrichment analysis was carried out in clusterProfiler^46^ and Metascape^47^ (http://metascape.org).

### Developmental pseudotime analysis and TF inference

The developmental pseudotime of MSCs was inferred using UMI count in Monocle.^48^ For the freshly isolated MSCs shown in Fig. 3a, since we had already obtained the marker genes of the two MSC clusters, we employed these genes to infer the developmental pseudotime. For all MSCs, combining both fresh and cultured MSCs, we followed “unsupervised ordering” in vignette to construct single-cell trajectories with default parameters.

For TF inference, we used SCENIC (https://scenic.aertslab.org/) to infer the gene regulatory networks from our scRNA-seq data and identify key TFs during the development of MSCs.^27^

### Cell sorting

Freshly harvested BMNCs were suspended in 4 °C HBSS + (Hanks-Balanced Salt Solution supplemented with 2% FBS, 10 mM HEPES, and 1% penicillin/streptomycin), followed by staining fluorochrome-conjugated or isotype control antibodies on ice for 30 min. The antibodies used in the present study were as follows: anti-CD45-APC (BioLegend, clone 2D1, 1:200), anti-CD45-Pacific Blue (BioLegend, clone 2D1, 1:200), anti-CD31-APC (BioLegend, clone WM59, 1:200), anti-CD31-Pacific Blue (BioLegend, clone WM59, 1:200), anti-CD235a-APC (Biolegend, clone HI264, 1:200), anti-CD235a-Pacific Blue (Biolegend, clone HI264, 1:200), anti-CD43-APC (Biolegend, clone CD43-10G7, 1:200), anti-CD44-PE (Biolegend, clone BJ18, 1:200), anti-CD73-APCcy7 (BioLegend, clone AD2, 1:200), anti-TM4SF1-FITC (Miltenyi Biotec, clone REA851, 1:200), anti-CD118-PE (BD, clone, 1:200), anti-CD140b (PDGFRβ)-APC (BioLegend, clone 18A2, 1:200), and anti-TM4SF1-Alexa Fluor 405 (RandD, FAB8164V, 1:100). Flow cytometry analysis and sorting were performed on a triple-laser MoFlo (Dako) or FACSCalibur (BD) flow cytometer, and data were analyzed using FlowJo software (Tree Star).

### Colony-forming unit fibroblast (CFU-F) assay

For CFU-F cultures, single cells were seeded into 96-well plates (1 single-cell/well) containing Mesenchymal Stem Cell Medium (ScienCell, US) supplemented with 1% Penicillin/Streptomycin solution, and incubated at 37 °C with 5% CO2. We then changed half of the medium every 3–4 days. When cultured for 2 weeks, the cells were fixed and stained with crystalline violet staining solution.

### Transplantation

Nude mouse transplantation was performed as described previously. Briefly, approximately 10^3^–10^4^ cells from single clones were mixed with β-TCP carrier (Bicon, Boston, MA, USA) and then subcutaneously implanted into the dorsal side of nude mice. Specimens were harvested at 4 and 8 weeks after transplantation, and the animals were killed by CO_2_ asphyxiation. The bone constructs were fixed in 4% paraformaldehyde and decalcified in 10% EDTA (pH 7.4) for 10 days. Finally, the specimens were dehydrated and then embedded in paraffin. All animal experiment conducted in the current study were approved by the Peking University Biomedical Ethics Committee on Experimental Animal Ethics.

### Histological staining

Bones tissues were fixed in 4% paraformaldehyde at 4 °C for 1–3 days by continuous agitation, and then decalcified at room temperature. Replace the fresh 14% EDTA solution every 24 h, in which EDTA was dissolved in Milli-Q water, and adjusted the pH value to 7.1 with ammonium hydroxide. After complete decalcification, the tissues were washed in PBS for 2 h, and then soaked in PBS containing 30% sucrose. Next, put the samples at 4 °C under constant agitation overnight and finally embedded in paraffin. Bone sections (5-μm thickness) were stained with hematoxylin and eosin (H&E) and Oil-red O staining.

## Supplementary information

Supplementary Materials

Supplementary Table 1

Supplementary Table 2

Supplementary Table 3

Supplementary Table 4

## Data Availability

All sequencing data in this work are stored in the Genome Sequence Archive (GSA) database (project number: PRJCA012045).
