# Supplementary material for: Characterization of mesenchymal stem cells in human fetal bone marrow by single-cell transcriptomic and functional analysis
Source: Signal Transduct Target Ther. 2023 Mar 31;8:126. doi: 10.1038/s41392-023-01338-2 (PMC10063684; doi:10.1038/s41392-023-01338-2)
Supplement: Supplementary file 1 — Supplementary Materials [file 41392_2023_1338_MOESM1_ESM.docx]

Supplementary Materials for

**Characterization of mesenchymal stem cells in human fetal bone marrow by single-cell transcriptomic and functional analysis**

Ping Zhang^1,3,8^, Ji Dong^4,8^, Xiaoying Fan^4,8^, Jun Yong^2,6^, Ming Yang^2,6^, Yunsong Liu^1,3^, Xiao Zhang^1,3^, Longwei Lv^1,3^, Lu Wen^1,4^, Jie Qiao^2,6,7^*, Fuchou Tang^2,5,7,^*, Yongsheng Zhou^1,3,^*

Correspondence to: [kqzhouysh@hsc.pku.edu.cn](mailto:kqzhouysh@hsc.pku.edu.cn) (Y.Z.), tangfuchou@pku.edu.cn (F.T.), jie.qiao@263.net (J.Q.)

**This PDF file includes:**

Supplementary Fig. 1 to 7

Supplementary Table 1 to 4


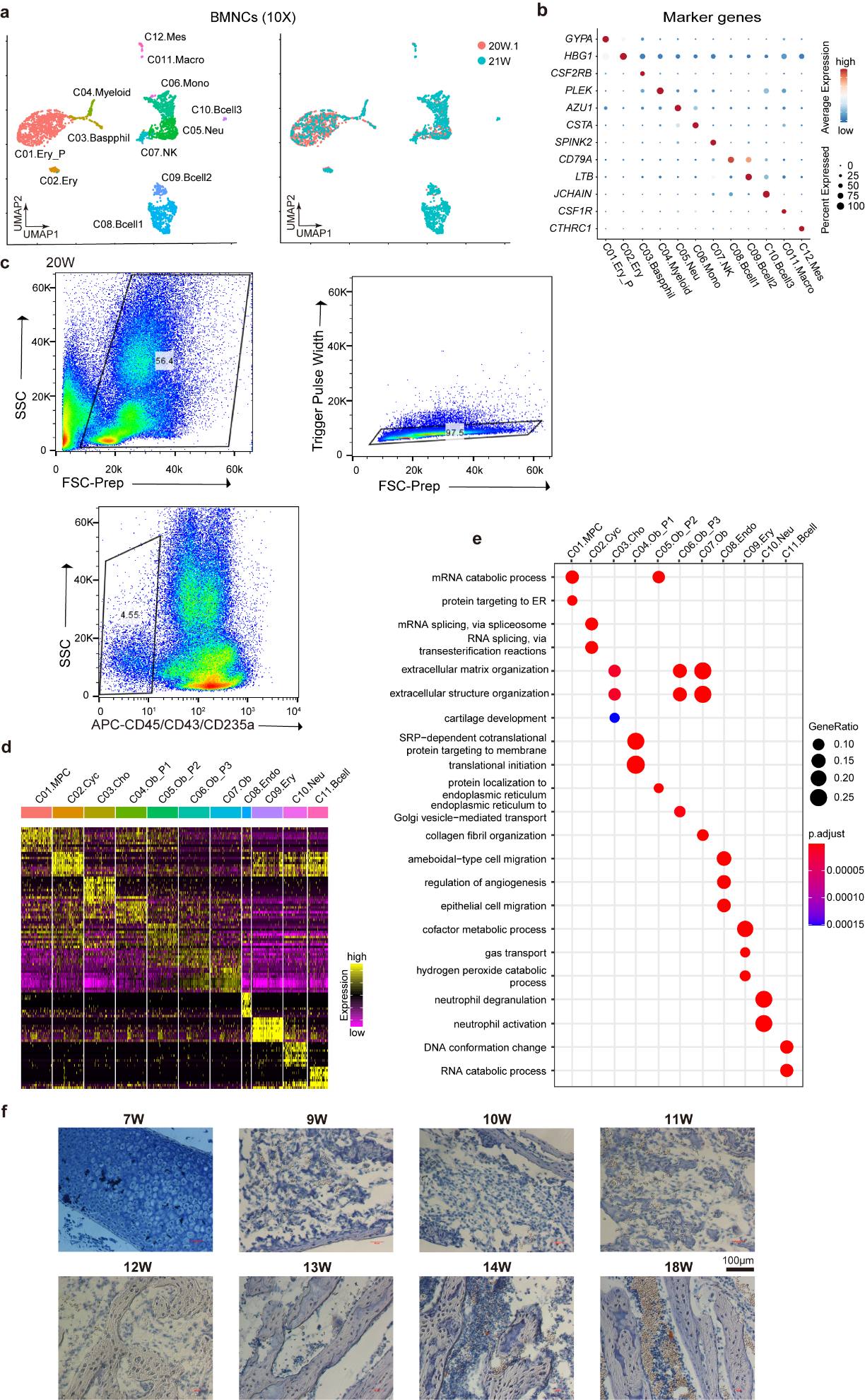


Supplementary Fig. 1. Expression landscape of human fetal BMNCs.

1. UMAP showing the clustering (left) and week information (right) of human fetal BMNCs without FACS sorting, which were sequenced by 10X Genomics scRNA-seq technique.
2. Dotplot showing the expression patterns of representative marker genes in each major cluster identified in Fig. S1a. The color key from blue to red indicates low to high expression levels, respectively. Dot size indicates the percent of cells expressing a certain gene.
3. Flow cytometry analysis showed that CD45^-^CD43^-^CD235a^-^ bone marrow nucleated cells using a 20-week-old embryo as an example.
4. Heatmap showing the DEGs of clusters identified in Fig. 1b. The color key from purple to yellow indicates low to high expression levels, respectively.
5. GO terms based on the DEGs of clusters identified in Fig. S1d.
6. Oil-red-O staining of femur sections from different embryonic stages.


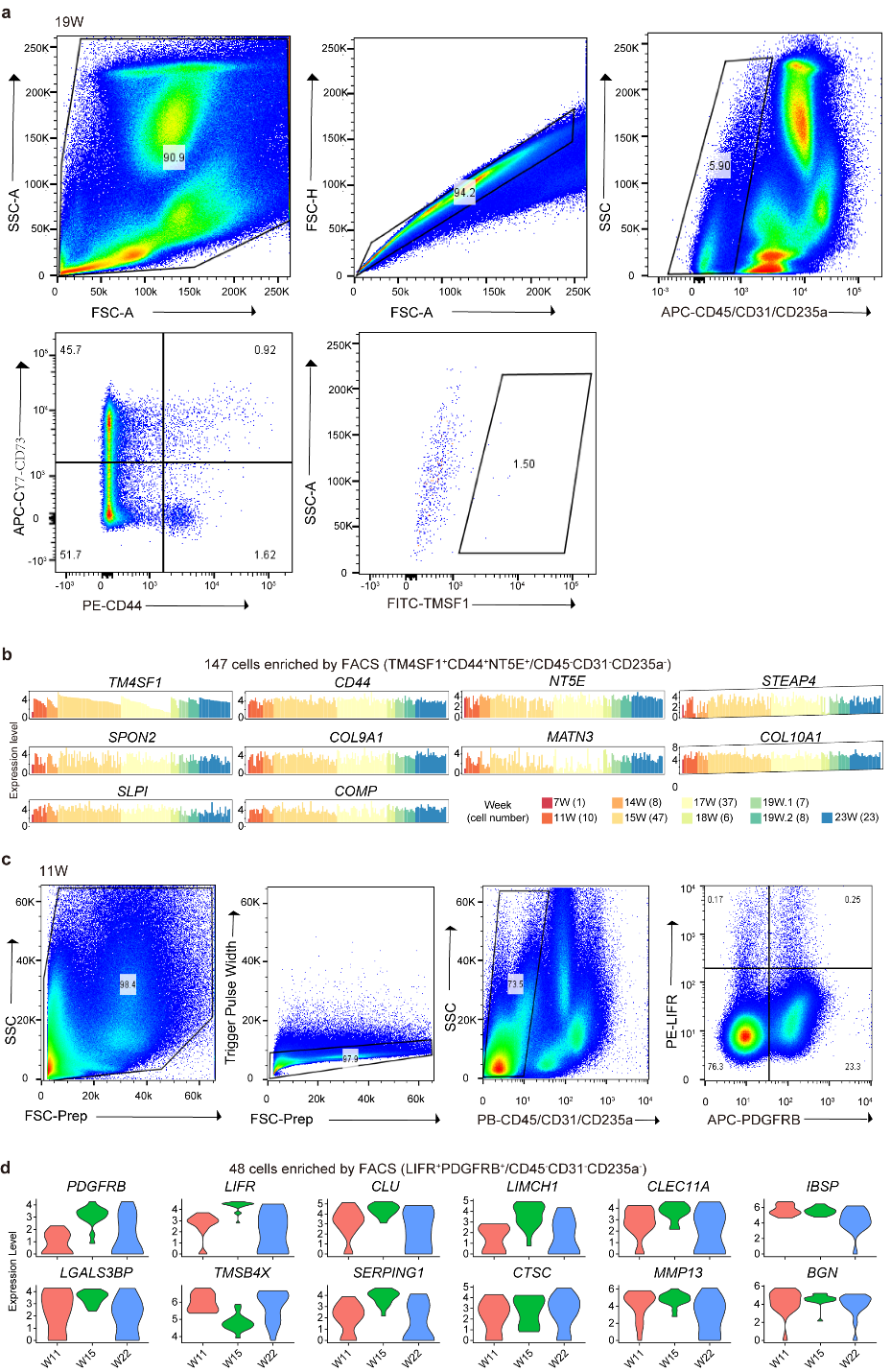


Supplementary Fig. 2. FACS sorting human fetal BM derived stem cells.

1. Flow cytometry analysis of TM4SF1^+^CD44^+^CD73^+^/CD45^-^CD31^-^CD235a^-^ BMNCs.
2. Barplot showing expression levels of representative marker genes of chondrocytes in TM4SF1^+^CD44^+^CD73^+^/CD45^-^CD31^-^CD235a^-^ BMNCs.
3. Flow cytometry analysis of LIFR^+^PDGFRB^+^/CD45^-^CD31^-^CD235a^-^ BMNCs.
4. Violin plots showing expression levels of representative marker genes of MSCs in LIFR^+^PDGFRB^+^/CD45^-^CD31^-^CD235a^-^ BMNCs.


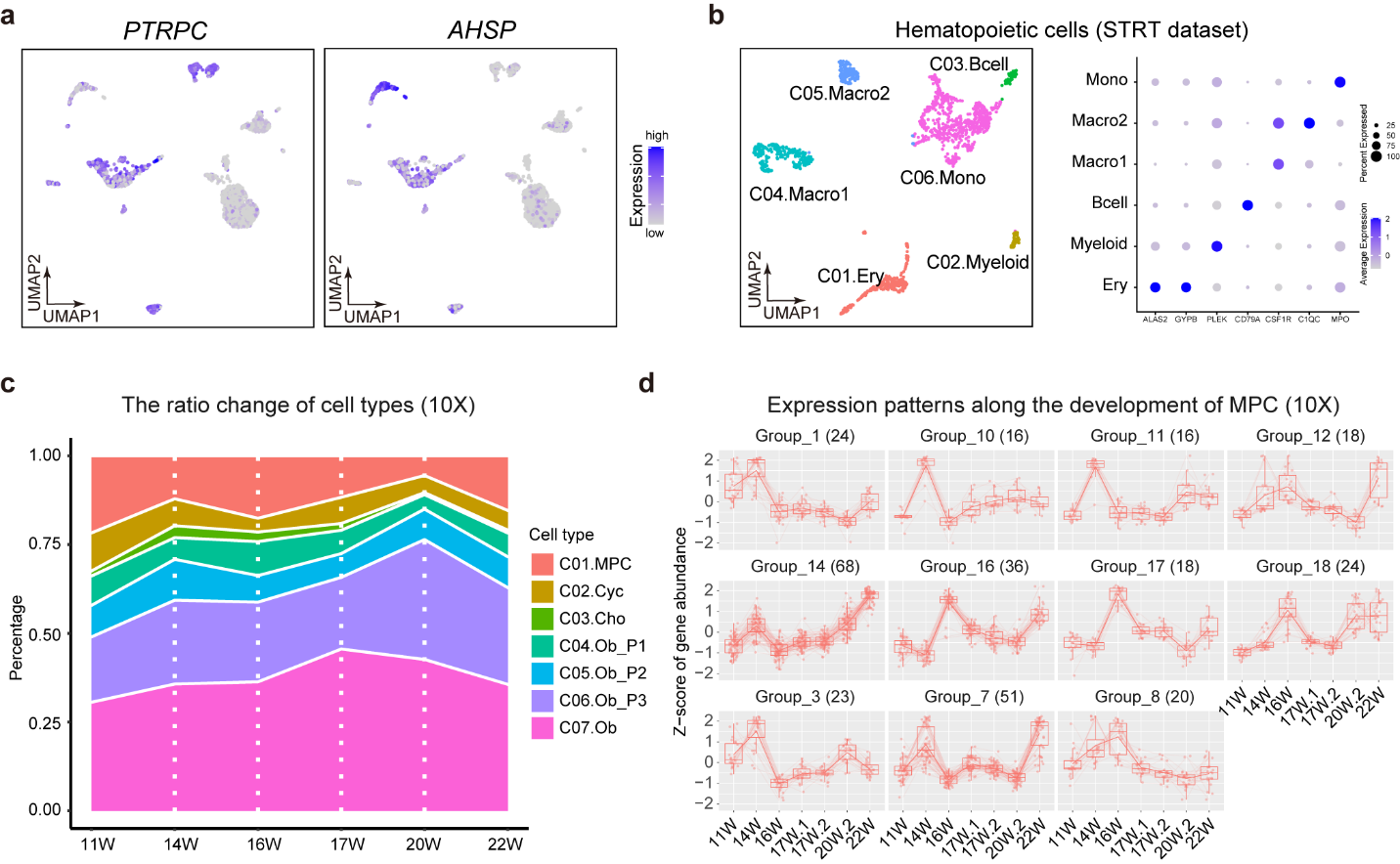


Supplementary Fig. 3. Expression landscape of human fetal BMNCs at early stages.

1. UMAP showing the marker genes of hematopoietic cells in human fetal BM by STRT scRNA-seq technique.
2. UMAP showing the clustering of hematopoietic cells in human fetal BM (left). Representative marker genes of each cluster are shown on the right.
3. The cell type ratio from 11 to 22 weeks.
4. Expression patterns along the development of MSCs. The gene number is in the bracket.


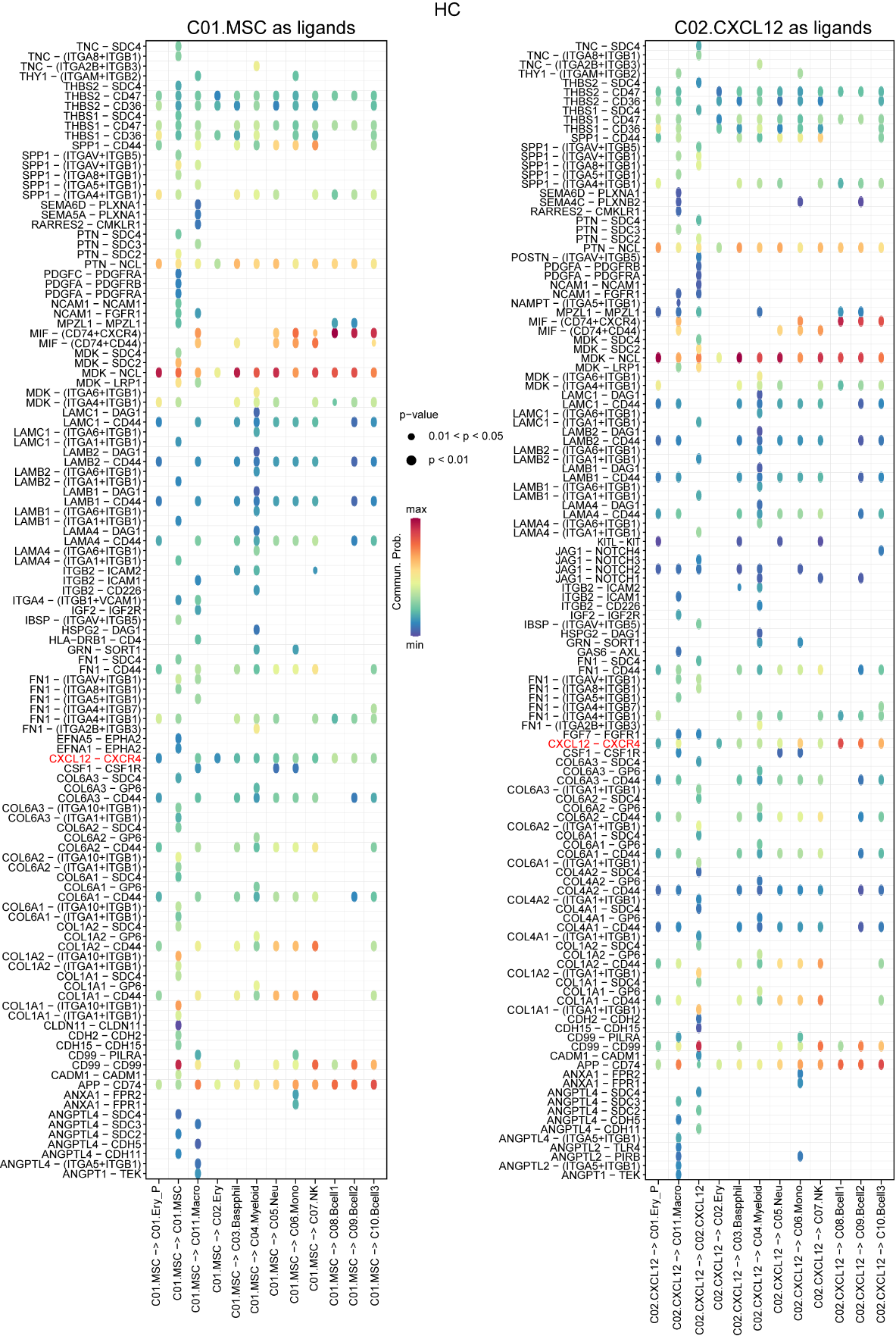


Supplementary Fig. 4. Cellular interaction between MSCs and hematopoietic cells (HC).

**
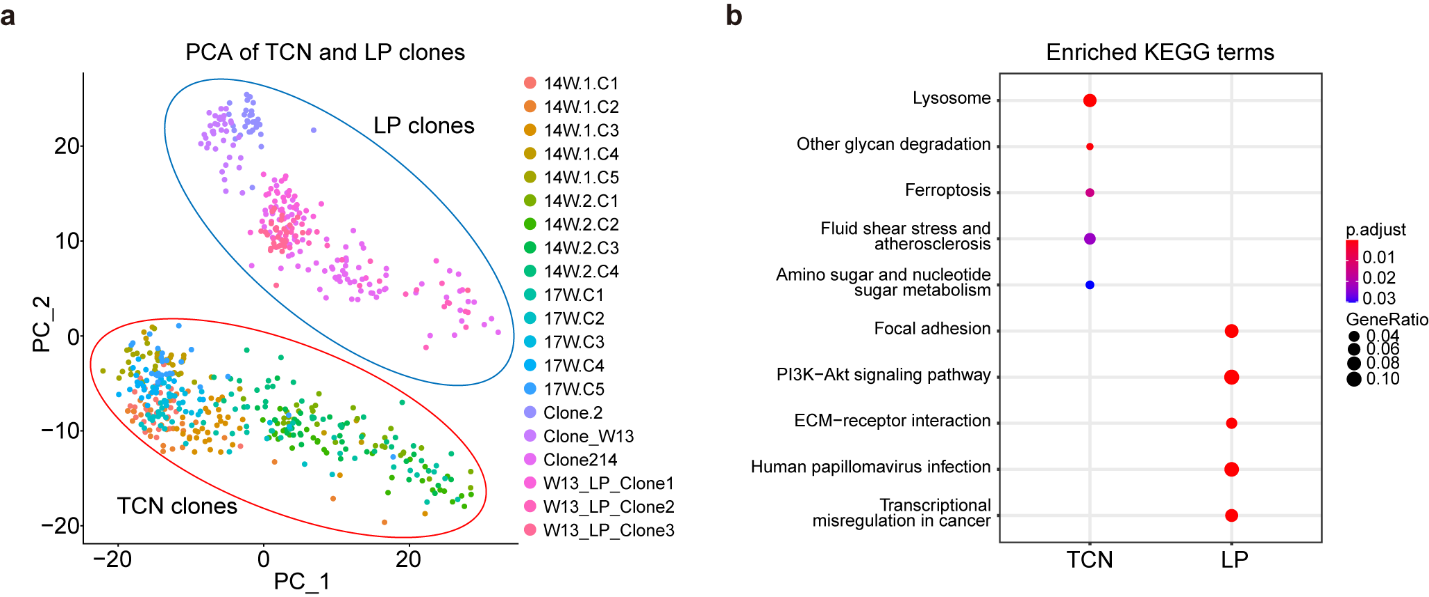
**

Supplementary Fig. 5. Comparison between TCN clones and LP clones.

1. PCA analysis exhibited distinct differences between TCN clones and LP clones.
2. Enriched KEGG terms using DEGs of TCN clones and LP clones.


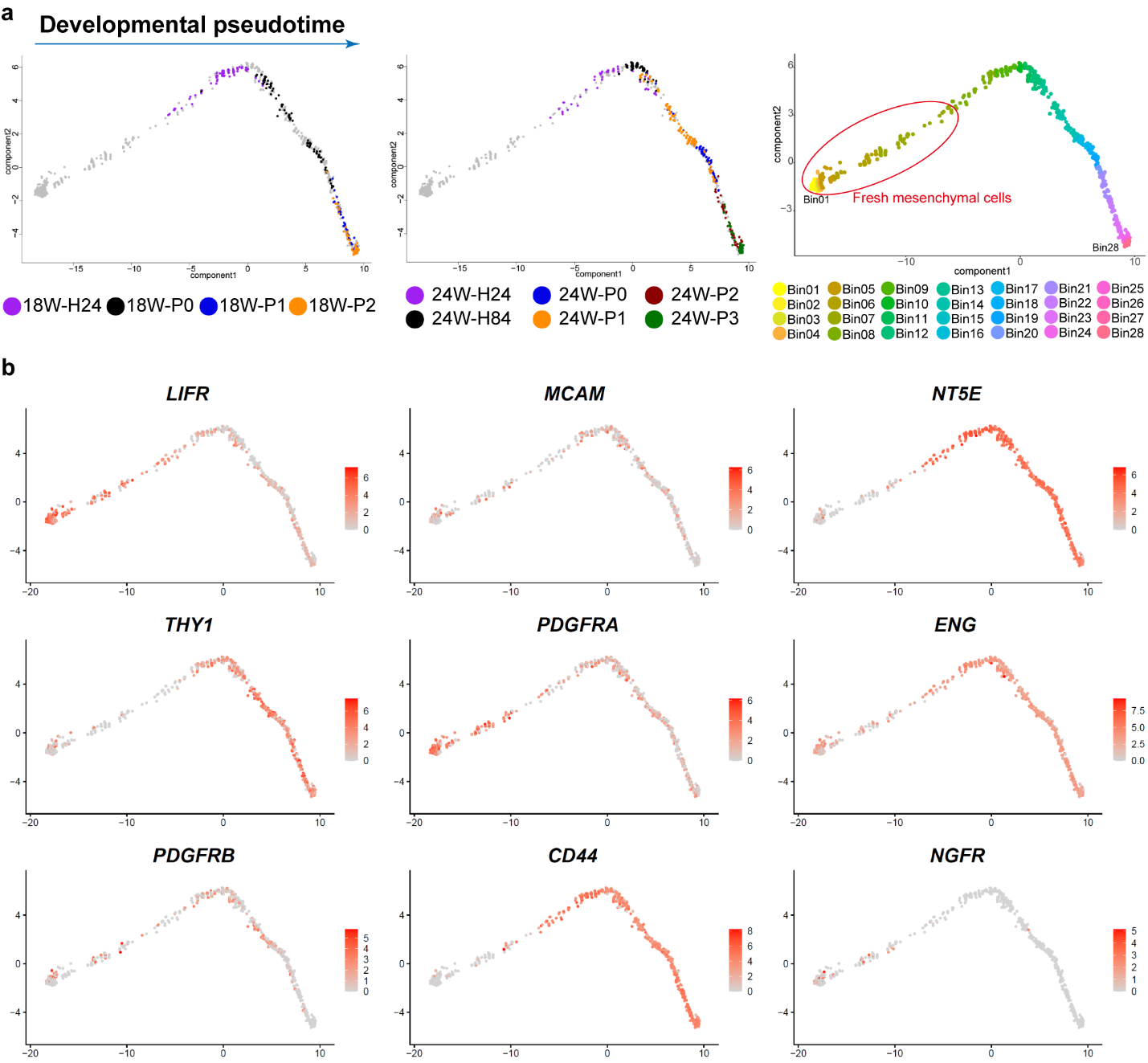


Supplementary Fig. 6. Comparison between cultured and fresh embryonic MSCs.

1. Temporal information for cultured mesenchymal cells mapped in the plots of developmental pseudotime (right and middle). Information for split bins mapped in the plots of developmental pseudotime (left).
2. The expression patterns of representative marker genes along the developmental pseudotime.


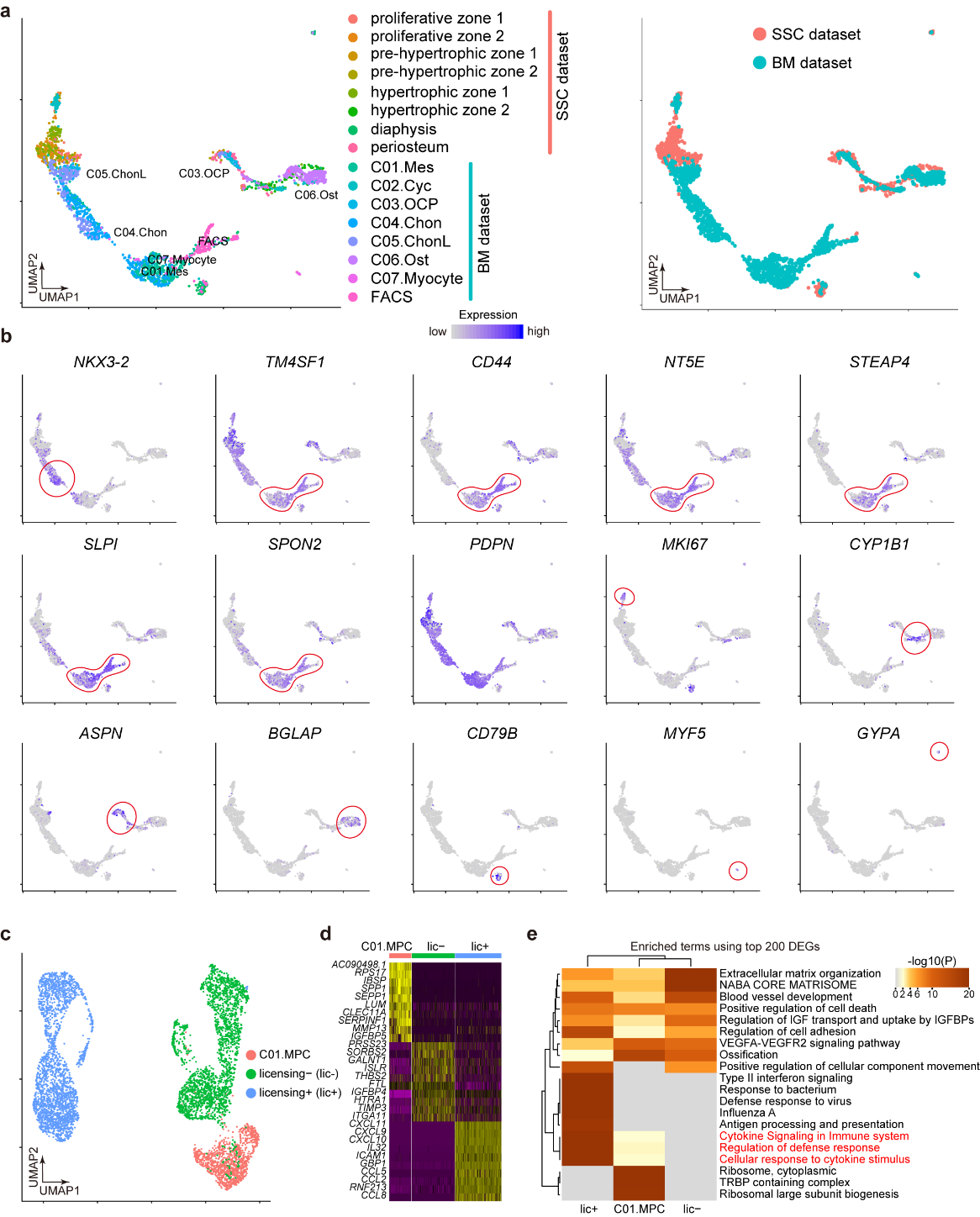


Supplementary Fig. 7. Comparison between BM mesenchymal cells and SSCs.

1. BM mesenchymal cells identified in our study were quite different from the human embryonic skeletal stem cells (SSCs) identified by Longaker group.
2. Expression levels of representative marker genes mapped on the UMAP plots. Marker genes related cell types were circled and displayed.

c-e. Distinct expression patterns among fetal, licensing+ and licensing- MSCs, as determined by UMAP, heatmap and Go term analysis.

**Supplementary Table Legends**

**Supplementary Table 1. Sample information**

**Supplementary Table 2. Differentially expressed genes in 10X and STRT datasets.**

**Supplementary Table 3. Cellular interactions between mesenchymal cells and hematopoietic cells.**

**Supplementary Table 4. Barcode information of STRT scRNA-seq.**
